# Infective Endocarditis by *Listeria* Species—A Systematic Review

**DOI:** 10.3390/jcm13195887

**Published:** 2024-10-02

**Authors:** Despoina Kypraiou, Maria Konstantaraki, Andreas G. Tsantes, Petros Ioannou

**Affiliations:** 1 School of Medicine, University of Crete, 71003 Heraklion, Greece; 2 Laboratory of Hematology and Blood Bank Unit, School of Medicine, “Attikon” University Hospital, National and Kapodistrian University of Athens, 12462 Athens, Greece; andreas.tsantes@yahoo.com

**Keywords:** *Listeria*, infective endocarditis, aortic valve, mitral valve

## Abstract

Infective endocarditis (IE) is a disease associated with significant morbidity and mortality. It is more commonly caused by Gram-positive cocci, but Gram-positive bacilli may seldom cause the disease. *Listeria monocytogenes* is an aerobic Gram-positive coccobacillus and a foodborne and opportunistic pathogen most commonly causing gastrointestinal infections, even though bacteremia, sepsis, meningitis, and fetal infections may also occur. *Listeria* IE has rarely been described, with most reports being case reports or case series. Thus, the characteristics of this disease remain largely unknown. This systematic review aimed to present all published *Listeria* IE studies and describe their characteristics. A search of PubMed, Scopus, and the Cochrane Library for studies providing information on epidemiology, clinical findings, treatment, and outcome of *Listeria* IE cases was performed. A total of 54 studies containing data from 62 patients were included. Among all patients, 64.5% were male; the median age was 69 years. Among all patients, 54.8% had a history of a prosthetic valve. The aortic valve was the most commonly affected, followed by the mitral. Fever, heart failure, and embolic phenomena were the most commonly encountered clinical findings. The only isolated species was *L. monocytogenes*. Antimicrobial resistance was relatively low for aminopenicillins and aminoglycosides, the most commonly used antimicrobials for treating *L. monocytogenes* IE. Surgery was performed in 27.4% of patients. Mortality was 37.1%. Patients who survived were more likely to have had a prosthetic valve, to have necessitated transesophageal echocardiography for the diagnosis, to have mitral valve IE, and to have had surgical management; however, no factor was identified in a multivariate logistic regression model as an independent factor for overall mortality.

## 1. Introduction

Infective endocarditis (IE) constitutes an infection that affects the endothelium of the heart, the heart valves (either native or prosthetic), and intracardiac devices. Although it remains a rare condition, with its incidence ranging between 6 and 24/100,000 person–years depending on the geographical area (e.g., age-standardized incidence rate is 13–18/100,000 person–years in Europe, 13–25/100,000 person–years in the Americas, 6–16/100,000 person–years in Asia, 12/100,000 person–years in Oceania, and 7–9/100,000 person–years in Africa) [1,2], it has been associated with high rates of morbidity and mortality, especially if not treated properly and promptly and can lead to a prolonged hospital stay. IE is associated with specific risk factors, such as underlying cardiac conditions, prosthetic cardiac material such as a cardiovascular implantable electronic device (CIED) or prosthetic cardiac valves, rheumatic disease, poor dental hygiene, and intravenous drug use (IVDU). Multiple clinical, imaging, and laboratory findings and the modified Duke’s criteria are used to diagnose IE. The most common (typical) etiological agents identified are Gram-positive cocci such as staphylococci, streptococci, and enterococci, accounting for almost 75% of all cases of IE [3,4]. Appropriate treatment includes several weeks of antibiotics, and in some cases of complicated IE, surgery might be required.

*Listeria monocytogenes* is an aerobic Gram-positive coccobacillus and a foodborne and opportunistic pathogen. It is commonly found in animal feed, soil, and almost any type of animal tissue or fluids, as well as raw and unprocessed food such as dairy products, vegetables, and meat [5,6]. It can invade various host cells, including phagocytes and epithelial and parenchymal cells, through phagocytosis and resides and multiplies within the cytoplasm, forming a comet-like structure. This process promotes the camouflage of the bacteria and protects them from the host’s immune defense procedures and the antibiotics present in the extracellular fluid [5,6]. In humans, *L*. *monocytogenes* can cause a wide spectrum of clinical manifestations in cases where host factors that favor invasive disease are present or when the bacteria load in the intestinal tract is high enough to surpass topical defenses of the gastrointestinal tract. Hence, it affects specific groups of people, including the elderly, pregnant women, fetuses, and immunocompromised individuals [5,6].

A rare but severe complication of *L. monocytogenes* bacteremia is IE, which occurs in native and prosthetic valves. Mortality rates of IE by this pathogen range between 37% and 50% [4]. As its diagnosis and management remain challenging, and reports on this infection are rather scarce, studies providing information on *L. monocytogenes* IE would be of great value.

Given the rarity of IE by *L. monocytogenes*, the scarce data in the literature, and the need for reliable guidance for the clinician should this condition occur, we aimed to systematically review all cases of *L. monocytogenes* IE and describe patient characteristics, treatment administered, infection outcomes, and mortality.

## 2. Materials and Methods

### 2.1. Search Strategy and Inclusion and Exclusion Criteria

For this review, we adopted the Meta-analysis of Observational Studies in Epidemiology (MOOSE) guidelines, which are more appropriate for systematic reviews assessing epidemiological studies [7]. Eligible studies were identified by searching PubMed, Scopus, and Cochrane Library with the following text words: ‘*Listeria* AND endocarditis’. All eligible studies published until 19 September 2024 were included in the further analysis.

The study’s primary outcomes were recording data on the baseline characteristics of patients with *Listeria* IE (gender and age) and data on patients’ outcomes. Secondary outcomes were recording data on the exact site of infection, the patients’ clinical characteristics, the antimicrobial susceptibility of the pathogens, and the treatment administered.

Studies were included in the analysis if they were in English and reported data on patients’ clinical characteristics, microbiology, treatment, and outcomes. Secondary research papers (e.g., reviews), editorials, and papers not reporting primary research in humans were excluded from the analysis. Additionally, studies not in English and those not referring to IE by *Listeria* were also excluded. Two investigators (MK, DK) using Rayyan independently reviewed the titles and abstracts of the resulting references and then retrieved and rescreened the full-text publications of potentially relevant articles [8]. Study selection was based on consensus. Reference lists of included studies were searched for relevant articles. When the investigators could not access a full-text publication, attempts were made to communicate with the study’s authors to kindly provide the full text.

### 2.2. Data Extraction and Definitions

Two investigators (DK, MK) extracted data from each eligible study. The extracted data included study type, year of publication, and country; patient demographic data (gender and age); patients’ medical history (previous cardiac surgery or cardiac valve replacement, time after cardiac valve replacement); infection data and microbiology (infection site, data regarding microorganism identification, complications, embolic phenomena); treatment administered; whether surgery was performed; and outcome (i.e., overall mortality). Data on microbiology and the association of mortality with the IE episode were reported based on the study’s authors. Diagnosis of IE was confirmed by the investigators based on information provided by the authors and the modified 2023 Duke-ISCVID criteria if the diagnosis was at least possible (i.e., having at least one major and one minor criterion or at least three minor criteria) or if adequate pathological data justified a diagnosis of IE [9]. The recorded complications included any organ dysfunction or clinical deterioration that the authors considered to be related to the IE episode. The quality of evidence of the included studies’ outcomes was assessed using the Grading of Recommendations Assessment, Development and Evaluation (GRADE) [10].

### 2.3. Statistical Analysis

Data are presented as numbers (%) for categorical variables and median (interquartile range, IQR) for continuous variables. Continuous variables were compared using the Mann–Whitney U-test for non-normally distributed variables or the *t*-test for normally distributed variables. All tests were two-tailed, and a *p*-value equal to or lower than 0.05 was considered significant. A univariate linear regression analysis was conducted to identify factors associated with the all-cause mortality of patients. More specifically, univariate logistic regression was performed to identify any association between gender, age, presence of prosthetic cardiac valve, poor dental hygiene or recent dental work, history of a previous episode of IE, history of rheumatic heart disease, location of the infection (mitral, aortic, tricuspid, pulmonary, or IE at multiple valves), presence of fever, embolic phenomena, sepsis, heart failure, antimicrobial treatment, and surgical management, with all-cause mortality. Statistics were calculated with GraphPad Prism 6.0 (GraphPad Software, Inc., San Diego, CA, USA). A multivariate logistic regression analysis evaluated the effect of factors previously identified in the univariate analysis model associated with all-cause mortality with a *p* < 0.1. Multivariate analysis was performed using the SPSS version 23.0 (IBM Corp., Armonk, NY, USA).

## 3. Results

### 3.1. Literature Search and Included Studies’ Characteristics

**Figure 1:**
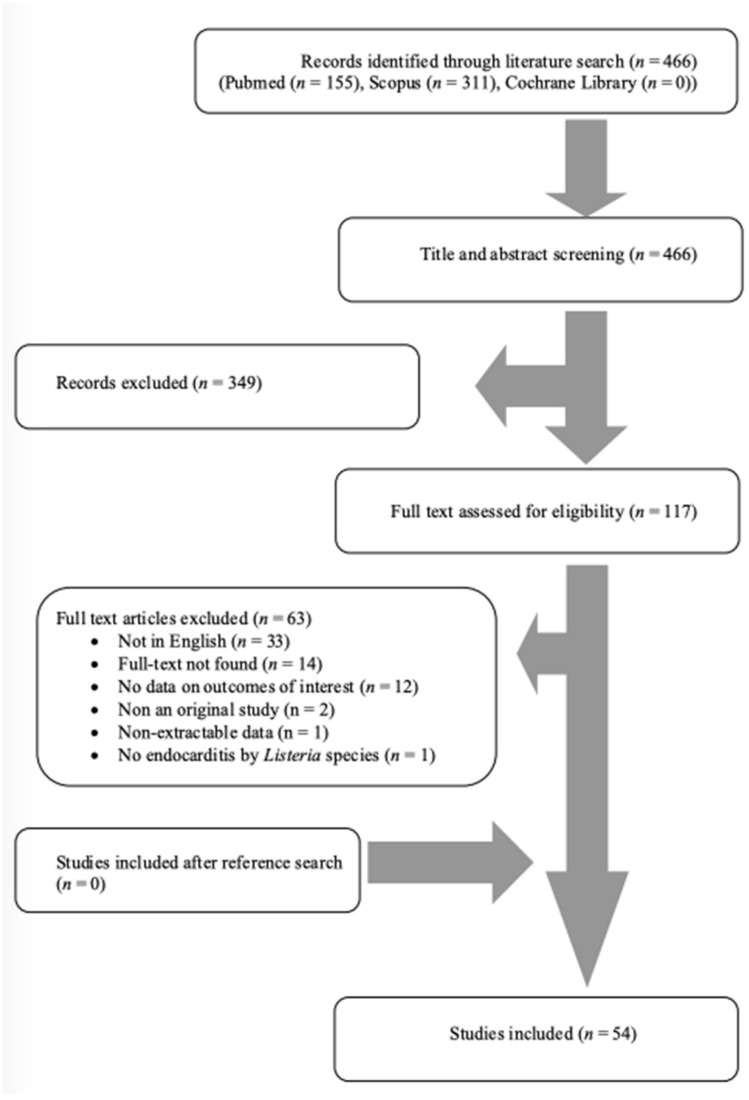
Flow diagram of study inclusion.

**Figure 2:**
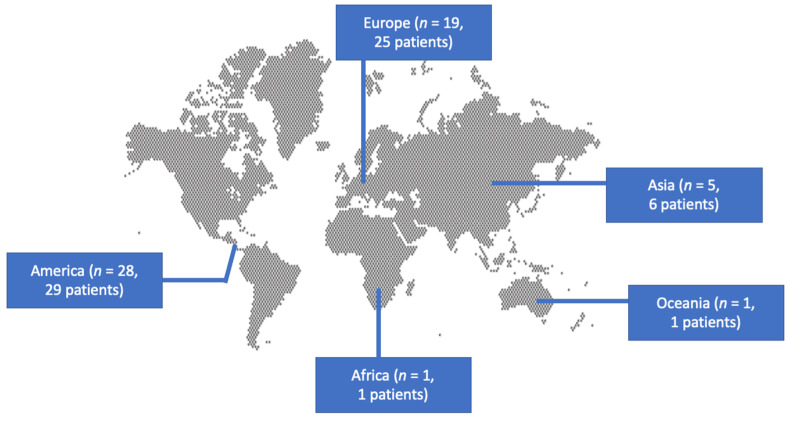
Geographical distribution of infective endocarditis by *Listeria* species.

A total of 466 articles from PubMed, Scopus, and Cochrane Library were screened. After reviewing the titles and abstracts, 117 articles were selected for full-text review. From these studies, 63 were excluded from the review: 33 articles were not in English, 14 articles could not be found, 12 articles did not have any outcomes of interest, 2 articles were non-original, 1 had non-extractable data, and, in 1 article, there was no case of *Listeria* IE. Additionally, no study was included after a reference search of the aforementioned studies. Finally, 54 met the present study’s inclusion criteria [5,11,12,13,14,15,16,17,18,19,20,21,22,23,24,25,26,27,28,29,30,31,32,33,34,35,36,37,38,39,40,41,42,43,44,45,46,47,48,49,50,51,52,53,54,55,56,57,58,59,60,61,62,63]. The review process is graphically presented in Figure 1. These studies provided data about 62 patients with *Listeria* species IE. Table 1 summarizes the characteristics of the included studies. Among them, 28 were conducted in North and South America, 19 in Europe, 5 in Asia, 1 in Oceania, and 1 in Africa. Figure 2 shows the geographical distribution of *Listeria* species IE cases. There were 49 case reports; thus, the overall quality of the evidence that contributed to this systematic review was rated as very low [10].

### 3.2. Basic Characteristics of Patients with Listeria IE

The median age of patients with *Listeria* ΙΕ was 69 years, ranging from 32 to 84 years, and 64.5% (40 out of 62) were male. A history of a prosthetic cardiac valve was noted in 54.8% (34 patients), rheumatic heart fever was noted in 13.6% (8 out of 59), congenital heart disease was noted in 5.1% (3 out of 59), and the presence of a CIED was noted in 9.7% (6 out of 62). The percentage of patients that had experienced IE in the past was 8.5% (5 out of 59 with available data), and 4.9% (3 out of 61 with available data) were diagnosed during the postoperative period after cardiac surgery. Notably, 8.6% (5 out of 58) had a possible exposure to dairy products, and 1.8% (1 out of 55) had poor dental hygiene or recent dental work. Table 1 shows the characteristics of patients with *Listeria* IE in detail.

### 3.3. Diagnostic Evaluation of Patients with Listeria IE

Blood cultures were positive in 98.4% (61 out of 62 patients). Infection was polymicrobial in 1.6% (1 patient), and the concomitantly isolated pathogen was *Staphylococcus aureus*. The only identified *Listeria* species was *L. monocytogenes*. The pathogen was isolated in the blood in 98.4% (61 patients) and in tissue cultures in 1.6% (1 patient).

Antimicrobial resistance was 33.3% (4 out of 12 strains in studies with available data) to aminoglycosides, 16.7% (2 out of 12) to ampicillin, 10% (1 out of 10) to trimethoprim and sulfamethoxazole, but 0% (0 out of 6) to quinolones.

A transthoracic echocardiogram was used for diagnosis in 40.7% (22 out of 54 patients with available data), while a transesophageal echocardiogram was used in 31.5% (17 out of 54). Most commonly, *Listeria* ΙΕ affected the aortic valve at rates up to 50% (27 out of 54 patients with available data). The next most frequently affected valve was the mitral, in 46.3% (25 out of 54), with fewer infections localized in multiple valves, the tricuspid valve, or CIED.

### 3.4. Clinical Characteristics of Patients with Listeria IE

Fever was present in 63.3% (38 out of 60 patients with available data), sepsis was noted in 41.4% (12 out of 29 patients), embolic phenomena were diagnosed in 51.4% (19 out of 37 patients), immunological phenomena were noted in 10.7% (3 out of 28 patients), and paravalvular abscesses were found in 25% (9 out of 36 patients). Table 2 provides detailed information on the diagnosis and clinical presentation of *Listeria* IE.

### 3.5. Treatment and Outcomes of Listeria IE

The detailed treatment provided for *Listeria* ΙΕ can be seen in Table 1 and is also summarized in Table 2. The most common antibiotic class administered was aminoglycosides in 60% (36 out of 60 patients with available data), while aminopenicillin was administered in 56.7% (34 patients). Surgical management, along with antimicrobials, was essential in 27.9%. All-cause mortality was 37.7% (23 out of 61) and was attributed directly to IE in 24.6% (15 out of 61).

### 3.6. Statistical Analysis of Listeria ΙΕ

A statistical comparison of patients with *Listeria* ΙΕ who survived with those who died revealed that those who survived were more likely to have had a prosthetic valve, to have necessitated transesophageal echocardiography for the diagnosis, to have mitral valve IE, and to have had surgical management. Additionally, those who survived were also less likely to have presented with shock. The statistical comparison results can be seen in Table 2. Moreover, a univariate linear regression analysis of overall mortality with several patients’ characteristics was performed and identified the presence of shock to be positively associated with overall mortality (*p* = 0.0140). The presence of a prosthetic valve, IE in the mitral valve, and surgical management were negatively associated with overall mortality (*p* = 0.0144, *p* = 0.0198, and *p* = 0.0106, respectively). A multivariate logistic regression analysis, after including all parameters with a *p* < 0.1 after excluding the parameters that yielded infinite results, thus affecting stability, identified only shock to be independently associated with overall mortality. Table 3 shows the regression analysis of overall mortality in *Listeria* IE patients.

## 4. Discussion

Herein, the characteristics of patients with *Listeria* IE are reviewed. Most patients suffering from this infection were males, most patients had a prosthetic valve, and the most commonly affected valve was the aortic. The most common clinical findings included fever, heart failure, and embolic phenomena. Aminoglycosides and beta-lactams were the most commonly used antimicrobials, while 37% of patients died.

Gram-positive cocci are the most common pathogens identified as causes of IE [3,4]. Other bacteria, such as Gram-negative or Gram-positive bacilli, are much less frequent causes of IE. However, such cases have seldom been reported [64,65]. *Listeria* is a Gram-positive bacterium that was first described in 1926 during an outbreak affecting guinea pigs and rabbits [66]. It was recognized as a cause of human disease and as a foodborne pathogen in the 1970s and 1980s, respectively [67]. Common infections caused by *Listeria* include gastroenteritis, which typically requires the ingestion of a highly contaminated food to cause disease in immunocompetent individuals, and bacterial sepsis and meningitis in immunosuppressed individuals, or fetal infection in pregnant women [68,69,70]. *L. monocytogenes*, the only species known to cause disease in humans, has many complex mechanisms of regulation and several diverse responses to stress that enable it to survive and initiate its virulent stage. Recent years have shed light on the mechanistic insights of its biology, host–pathogen interaction, immune evasion, and pathogenesis with tools such as genomics, epigenomics, transcriptomics, and proteomics [68]. For example, bacterial cells can enter human cells, escape from the vacuole, change organelle morphology and function, manipulate host–cell transcriptional and epigenetic regulation, and spread from cell to cell by manipulating the host–cell cytoskeleton [68]. Notably, IE by *L. monocytogenes* is a rare condition with scarce data in the literature.

### 4.1. Comparison of Basic Characteristics among Patients with Listeria IE and IE by Other Species

The median age of patients with *Listeria* species IE in the present review was 69 years, which is close to that in other cohorts of patients with IE, where the mean age is about 70 years [3,4,71]. A male predominance was noted, as is the case in patients with IE in general [4,71]. A prosthetic valve was noted in 55% of patients with *L. monocytogenes* IE, which is close to the rate noted in studies of IE that can be up to 50% [3,4,71]. A previous episode of IE was noted in 8.5% of patients with IE by *L. monocytogenes*, and a history of rheumatic fever was noted in 13.6%. Both rates were comparable to those noted in patients with IE [3,71]. Congenital heart disease was noted in 5.1% of patients with *L. monocytogenes*, a rate similar to that in patients with IE in the general population [3].

Notably, possible exposure through dairy products was noted in 8.6% of patients with *L. monocytogenes* IE. This is a unique characteristic of this pathogen, among others, causing IE. The ability of *L. monocytogenes* to cause foodborne disease has been well documented. It is thought to be associated with its ability to grow in many different environmental conditions, such as in low-pH and high-salt concentrations and refrigeration temperatures, thus allowing for the pathogen to overcome common food preservation conditions and pose risks for human health [72].

The aortic was the most commonly infected valve, followed by the mitral. This is in accordance with other studies of IE [4,71]. Regarding clinical presentation, fever was the most common symptom in 63.3% of patients, heart failure in 53.8%, and embolic phenomena in 51.4%. The corresponding rates in other studies of IE in general were 84%, 33–52%, and 15–45%, respectively [3,4,71].

### 4.2. Antimicrobial Resistance of Listeria Species

Regarding antimicrobial resistance, strains in studies with available data were resistant to aminoglycosides in 33%, while the resistance rates to ampicillin and carbapenems were lower. Notably, antimicrobial resistance to trimethoprim and sulfamethoxazole was low, and resistance to quinolones was zero. In a recent study from Italy providing data on the antimicrobial resistance of *L. monocytogenes* strains isolated in food and food-processing environments, antimicrobial resistance to ampicillin, meropenem, and trimethoprim with sulfamethoxazole was 45%, 13%, and 37.5%, respectively [73]. Another recent study that evaluated the antimicrobial resistance of *L. monocytogenes* from ready-to-eat foods in South Africa identified less than 10% resistance to ampicillin, less than 20% resistance to imipenem, less than 20% resistance to trimethoprim and sulfamethoxazole, about 25% resistance to aminoglycosides, and about 30% resistance to quinolones [74]. Another recent study that evaluated thousands of clinical and food isolates identified minimal resistance to ampicillin and aminoglycosides [75]. The differences in antimicrobial resistance between these studies could be associated with the different geographical regions where the isolates were identified. Moreover, some of the different rates noted in the present review may be associated with the fact that few studies included in this study provided information regarding antimicrobial resistance. Moura et al. also evaluated the genotypic profile of resistant *L. monocytogenes* strains and identified both chromosomal (tetM) and plasmid-associated acquired resistance genes (lnuG, mphB). Acquired antimicrobial phenotypes were towards trimethoprim (dfrD), tetracyclines (mostly due to tetM), macrolides (ermB, mphB), lincosamides (lnuG), and phenicols (fexA). Eventually, most of the studies, including the present review, underline the need for antimicrobial resistance surveillance in *L. monocytogenes* strains, especially in serious infections, such as IE. However, aminopenicillins and aminoglycosides remain a viable choice in most cases of *L. monocytogenes* infections.

### 4.3. Comparison of Outcome among Patients with Listeria IE and IE by Other Species

Mortality was high, with more than one out of three patients dying, and most of those who succumbed did so due to *L. monocytogenes* IE. This rate was within the rate noted in other studies of IE in the general population, which was 11–40% [3,4,71]. In the current study, those who died revealed that those who survived were more likely to have had a prosthetic valve, to have necessitated transesophageal echocardiography for the diagnosis, to have mitral valve IE, and to have had surgical management. However, no such factor was identified from a multivariate logistic regression analysis as an independent factor for overall mortality.

### 4.4. Limitations

This review has some important limitations. First, it mainly includes information derived from case reports. Thus, the present results should be read cautiously, as the quality of evidence was low. Additionally, the credibility of the information included relies on the recording and reporting of each study’s authors. Moreover, the number of the included patients is also low, not permitting, thus, to derive too safe conclusions. Finally, publication bias may have affected the results presented in the present review.

### 4.5. Future Directions

Even though this infection is rare, a better understanding of it would be critical for clinicians caring for such patients. Thus, future multicenter prospective studies evaluating this condition in cohorts of patients would allow for drawing safer conclusions, excluding several limitations associated with the bias introduced by smaller retrospective studies and case reports. Such registries would be valuable both for *L. monocytogenes* IE and for IE by other rare bacteria.

## 5. Conclusions

To conclude, this systematic review presents the epidemiological, clinical, and microbiological characteristics of patients with *L. monocytogenes* IE, as well as information on the treatment and outcomes. *L. monocytogenes* was the only identified species. Possible exposure to dairy products was relatively infrequently reported. Antimicrobial resistance was low; thus, many antimicrobial options exist for treating this infection. Aminoglycosides and beta-lactams, commonly in combination, were used for treating this infection. Mortality was high and was mainly attributed to the episode of IE.

## Figures and Tables

**Table 1:** Characteristics of the included studies.

Study	Number of Patients	Age (Years)	Gender	Site of Infection *n* (%)	Treatment Administered, *n*	Infection Outcomes, *n* (%)
Hoeprich et al., 1955 [11]	1	42	Μale	NR	Penicillin	Deaths overall 0
Buchner et al., 1968 [12]	1	49	Μale	MV	Penicillin Aminoglycoside Tetracycline	Deaths overall 1 Deaths due to IE 1
De Soldati et al., 1972 [13]	2	32, 69	2 males	NR 1 (50) AoV 1 (50) TrV 1 (50)	Penicillin 1 (50) Aminopenicillin 1 (50) Tetracycline 1 (50) Chloramphenicol 1 (50)	Deaths overall 1 (50) Deaths due to IE 1 (50)
Bassan et al., 1975 [14]	1	75	Μale	NR	Penicillin	Deaths overall 0
Weinstein et al., 1976 [15]	1	72	Female	AoV	Penicillin Aminoglycoside	Deaths overall 1 Deaths due to IE 1
Clark et al., 1977 [16]	1	67	Female	AoV	Cephalosporin	Deaths overall 1 Deaths due to IE 1
Pitcher et al., 1978 [17]	1	53	Μale	NR	Penicillin	Deaths overall 0
Breyer et al., 1978 [18]	1	64	Μale	AoV	Penicillin	Deaths overall 1 Deaths due to IE 0
Saravolatz et al., 1978 [19]	1	58	Female	NR	Penicillin Aminoglycoside	Deaths overall 0
Gelber et al., 1980 [20]	1	66	Μale	NR	Penicillin Aminoglycoside	Deaths overall 0
Davis et al., 1983 [21]	1	69	Female	MV	Aminopenicillin Aminoglycoside	Deaths overall 0
Higgins et al., 1983 [22]	1	63	Μale	MV	Aminopenicillin Aminoglycoside	Deaths overall 0
Sheinman et al., 1985 [23]	1	74	Μale	NR	Aminopenicillin Aminoglycoside	Deaths overall 1 Deaths due to IE 0
Gallagher et al., 1986 [24]	1	69	Female	AoV	Vancomycin	Deaths overall 1 Deaths due to IE 0
Riancho et al., 1988 [25]	1	55	Μale	AoV MV	Penicillin Aminoglycoside	Deaths overall 0
Carvajal et al., 1988 [26]	3	58, 75, 83	1 male 2 females	AoV 3 (100)	Aminopenicillin 3 (100) Aminoglycoside 2 (66.7)	Deaths overall 3 (100) Deaths due to IE 3 (100)
Rao et al., 1989 [27]	1	73	Female	MV	Aminopenicillin Aminoglycoside	Deaths overall 0
Baddour et al., 1989 [28]	1	70	Female	NR	Aminopenicillin Aminoglycoside	Deaths overall 0
Hadorn et al., 1993 [29]	1	56	Female	MV	Trimethoprim–sulfamethoxazole	Deaths overall 0
Speeleveld et al., 1994 [30]	1	71	Μale	AoV	Aminopenicillin Aminoglycoside	Deaths overall 0
Castrocabezas et al., 1996 [31]	1	69	Μale	AoV	Vancomycin Aminoglycoside	Deaths overall 0
Danielsson-Tham et al., 1997 [32]	1	65	Μale	AoV	Penicillin	Deaths overall 1 Deaths due to IE 1
Manso et al., 1997 [33]	1	65	Μale	TrV	Aminopenicillin Aminoglycoside	Deaths overall 1 Deaths due to IE 1
Bemer-Meclhior et al., 1997 [34]	1	67	Female	AoV	Vancomycin Aminoglycoside Trimethoprim–sulfamethoxazole	Deaths overall 0
Johnston et al., 1998 [35]	1	76	Female	MV	Aminopenicillin Aminoglycoside	Deaths overall 1 Deaths due to IE 1
Raveh et al., 1998 [36]	2	63, 73	2 females	MV 2 (100)	Aminopenicillin 2 (100) Aminoglycoside 2 (100)	Deaths overall 0
Avery et al., 1999 [37]	1	41	Female	TrV	Penicillin Aminoglycoside Aminopenicillin	Deaths overall 1 Deaths due to IE 0
Benes et al., 2002 [38]	1	69	Female	MV	Aminopenicillin Trimethoprim–sulfamethoxazole	Deaths overall 0
Makaryus et al., 2004 [39]	1	81	Μale	AoV	Aminopenicillin Aminoglycoside	Deaths overall 0
Fernández Guerrero et al., 2004 [40]	2	68, 77	Μale	AoV 1 (50) MV 1 (50)	Penicillin 1 (50) Vancomycin 1 (50) Aminoglycoside 2 (100)	Deaths overall 0
Kida et al., 2006 [41]	1	74	Μale	MV	Penicillin	Deaths overall 0
Muñoz et al., 2006 [42]	1	76	Μale	AoV MV	Linezolid	Deaths overall 0
Karavidas et al., 2007 [43]	1	74	Μale	AoV	Carbapenem Aminoglycoside	Deaths overall 0
Llanwarne et al., 2007 [44]	1	76	Μale	MV	NR	Deaths overall 1 Deaths due to IE 1
Pocar et al., 2009 [45]	1	67	Μale	MV	Linezolid Trimethoprim–sulfamethoxazole	Deaths overall 0
Summa et al., 2010 [5]	1	78	Female	CIED	Aminopenicillin Aminoglycoside	Deaths overall 1 Deaths due to IE 1
Kelesidis et al., 2010 [46]	1	42	Female	AoV	Aminopenicillin	Deaths overall 0
Uehara Yonekawa et al., 2014 [47]	1	73	Female	Right atrium	Aminopenicillin Aminoglycoside	Deaths overall 1 Deaths due to IE 0
García-Granja et al., 2016 [48]	4	79, 80, 84, 84	3 males 1 female	MV 4 (100) AoV 1 (25)	Aminopenicillin 3 (75) Aminoglycoside 3 (75) Carbapenem 1 (25) Daptomycin 1 (25)	Deaths overall 2 (50) Deaths due to IE 0 (0)
Jyothidasan et al., 2016 [49]	1	54	Μale	TrV	Aminopenicillin Aminoglycoside	Deaths overall 0
Ciceri et al., 2017 [50]	1	66	Μale	AoV	Carbapenem Aminoglycoside	Deaths overall 0
Rahmati et al., 2017 [51]	1	83	Μale	AoV	Aminopenicillin Aminoglycoside	Deaths overall 0
Valckx et al., 2017 [52]	1	74	Μale	AoV	Aminopenicillin	Deaths overall 1 Deaths due to IE 1
Hasan et al., 2017 [53]	1	63	Μale	AoV	Penicillin Aminopenicillin	Deaths overall 0
Sharma et al., 2018 [54]	1	74	Μale	CIED	Aminopenicillin Vancomycin	Deaths overall 0
Kumaraswamy et al., 2018 [55]	1	79	Μale	AoV	Aminopenicillin	Deaths overall 1 Deaths due to IE 0
Shobayo et al., 2019 [56]	1	66	Female	AoV MV	Aminopenicillin Aminoglycoside	Deaths overall 0
Zhao et al., 2020 [57]	1	43	Μale	MV	Quinolone Teicoplanin	Deaths overall 0
Scheggi et al., 2021 [58]	1	32	Female	AoV	Aminopenicillin Linezolid Quinolone	Deaths overall 1 Deaths due to IE 1
Ogunleye et al., 2021 [59]	1	70	Μale	MV	Aminopenicillin Aminoglycoside	Deaths overall 0
Badar et al., 2022 [60]	1	77	Μale	MV	Aminopenicillin	Deaths overall 0
Randrianarisoa et al., 2022 [61]	1	75	Μale	AoV	Aminopenicillin Aminoglycoside	Deaths overall 0
Mohan et al., 2023 [62]	1	56	Μale	MV	Penicillin Aminoglycoside	Deaths overall 1 Deaths due to IE 1
Ramos-Ospina et al., 2024 [63]	1	54	Female	MV	Aminopenicillin Trimethoprim–sulfamethoxazole	Deaths overall 0

AoV: aortic valve; CIED: cardiac implantable electronic device; MV: mitral valve; TrV: tricuspid valve.

**Table 2:** Patients’ characteristics and infections’ outcomes.

Characteristic	All Patients (*n* = 62) *	Survived (*n* = 39) *	Died (*n* = 23) *	*p*-Value
Age, years, median (IQR)	69 (61.8–75)	69 (58–74)	72 (64–76)	0.3984
Male gender, *n* (%)	40 (64.5)	28 (71.8)	12 (52.2)	0.1701
Predisposing factors				
Prosthetic valve, *n* (%)	34 (54.8)	26 (66.7)	8 (34.8)	0.0357
Congenital heart disease, *n* (%)	3/59 (5.1)	1/38 (2.6)	2/21 (9.5)	0.2864
Possible exposure through dairy products, *n* (%)	5/58 (8.6)	3/38 (7.9)	2/20 (10)	1
Poor dental hygiene or recent dental work, *n* (%)	1/55 (1.8)	1/36 (2.8)	0/19 (0)	1
CIED, *n* (%)	6 (9.7)	5 (12.8)	1 (4.3)	0.3982
Previous IE, *n* (%)	5/59 (8.5)	3/38 (7.9)	2/21 (9.5)	1
Rheumatic fever, *n* (%)	8/59 (13.6)	7/38 (18.4)	1/21 (4.8)	0.2383
Central venous catheter, *n* (%)	2/59 (3.4)	0/38 (0)	2/21 (9.5)	0.1227
Post cardiac surgery, *n* (%)	3/61 (4.9)	3/38 (7.9)	0 (0)	0.2836
Method of diagnosis				
Transthoracic echocardiography, *n* (%)	22/54 (40.7)	13/34 (38.2)	9/20 (45)	0.7753
Transesophageal echocardiography, *n* (%)	17/54 (31.5)	15/34 (44.1)	2/20 (10)	0.0141
Valve localization				
Aortic valve, *n* (%)	27/54 (50)	14/32 (43.8)	13/22 (59.1)	0.4064
Mitral valve, *n* (%)	25/54 (46.3)	19/32 (59.4)	6/22 (27.3)	0.0275
Tricuspid valve, *n* (%)	4/54 (7.4)	1/32 (3.1)	3/22 (13.6)	0.2927
Multiple valves, *n* (%)	5/54 (9.3)	3/32 (9.4)	2/22 (9.1)	1
CIED, *n* (%)	2/56 (3.6)	1/34 (2.9)	1/22 (4.5)	1
Clinical characteristics				
Fever, *n* (%)	38/60 (62.7)	25/38 (65.8)	13/22 (59.1)	0.7815
Sepsis, *n* (%)	12/29 (41.4)	5/15 (33.3)	7/14 (50)	0.4621
Heart failure, *n* (%)	21/39 (53.8)	9/22 (40.9)	12/17 (70.6)	0.1059
Shock, *n* (%)	11/35 (31.4)	2/17 (11.8)	9/18 (50)	0.0040
Embolic phenomena, *n* (%)	19/37 (51.4)	13/22 (59.1)	6/15 (40)	0.3245
Paravalvular abscess, *n* (%)	9/36 (25)	5/20 (25)	4/16 (25)	1
Immunological phenomena, *n* (%)	3/28 (10.7)	0/15 (0)	3/13 (23.1)	0.0873
Treatment				
Aminoglycoside, *n* (%)	36/61 (59)	23 (59)	13/22 (59.1)	0.1348
Aminopenicillin, *n* (%)	35/61 (57.4)	20 (51.3)	15/22 (68.2)	1
Penicillin, *n* (%)	16/61 (26.2)	9 (23.1)	7/22 (31.8)	1
Carbapenem, *n* (%)	3/61 (4.9)	3 (7.7)	0/22 (0)	0.2494
Quinolone, *n* (%)	2/61 (3.3)	1 (2.6)	1/22 (4.5)	1
Vancomycin, *n* (%)	5/61 (8.2)	4 (10.3)	1/22 (4.5)	0.3745
Linezolid, *n* (%)	3/61 (4.9)	2 (5.1)	1/22 (4.5)	1
TMP-SMX, *n* (%)	5/61 (8.2)	5 (12.8)	0/22 (0)	0.0618
Surgical management, *n* (%)	17 (27.4)	15 (38.5)	2 (8.7)	0.0170
Outcomes				
Deaths due to infection, *n* (%)	15 (24.2)	NA	NA	NA
Deaths overall, *n* (%)	23 (37.1)	NA	NA	NA

CIED: cardiac implanted electronic device; IE: infective endocarditis; IQR: interquartile range; NA: not applicable; TMP-SMX: trimethoprim–sulfamethoxazole. *: data are among the number of patients mentioned on top unless otherwise described.

**Table 3:** Regression analysis of mortality in patients with *Listeria* infective endocarditis.

Characteristic	Univariate Analysis *p*-Value	Multivariate Analysis *p*-Value	OR (95% CI)
Mitral valve	0.0198	0.207	0.226 (0.034–2.078)
Heart failure	0.0681	0.214	4.074 (0.445–37.253)
Shock	0.0140	0.245	5.949 (0.295–120.098)
Surgical treatment	0.0106	0.032	0.052 (0.004–0.774)

CI: confidence interval; ORs: odds ratio.

## Data Availability

Not applicable.
